# Symbiotic Interactions Between Mosquitoes and Mosquito Viruses

**DOI:** 10.3389/fcimb.2021.694020

**Published:** 2021-08-30

**Authors:** Mine Altinli, Esther Schnettler, Mathieu Sicard

**Affiliations:** ^1^Molecular Entomology, Bernhard-Nocht-Institute for Tropical Medicine, Hamburg, Germany; ^2^German Centre for Infection Research (DZIF), Partner Site Hamburg-Luebeck-Borstel-Riems, Hamburg, Germany; ^3^ISEM, Université de Montpellier, CNRS, IRD, EPHE, Montpellier, France; ^4^Faculty of Mathematics, Informatics and Natural Sciences, University Hamburg, Hamburg, Germany

**Keywords:** mosquitoes, microbiota, *Wolbachia*, insect symbiosis, interactions, insect-specific viruses

## Abstract

Mosquitoes not only transmit human and veterinary pathogens called arboviruses (arthropod-borne viruses) but also harbor mosquito-associated insect-specific viruses (mosquito viruses) that cannot infect vertebrates. In the past, studies investigating mosquito viruses mainly focused on highly pathogenic interactions that were easier to detect than those without visible symptoms. However, the recent advances in viral metagenomics have highlighted the abundance and diversity of viruses which do not generate mass mortality in host populations. Over the last decade, this has facilitated the rapid growth of virus discovery in mosquitoes. The circumstances around the discovery of mosquito viruses greatly affected how they have been studied so far. While earlier research mainly focused on the pathogenesis caused by DNA and some double-stranded RNA viruses during larval stages, more recently discovered single-stranded RNA mosquito viruses were heavily studied for their putative interference with arboviruses in female adults. Thus, many aspects of mosquito virus interactions with their hosts and host-microbiota are still unknown. In this context, considering mosquito viruses as endosymbionts can help to identify novel research areas, in particular in relation to their long-term interactions with their hosts (e.g. relationships during all life stages, the stability of the associations at evolutionary scales, transmission routes and virulence evolution) and the possible context-dependent range of interactions (i.e. beneficial to antagonistic). Here, we review the symbiotic interactions of mosquito viruses considering different aspects of their ecology, such as transmission, host specificity, host immune system and interactions with other symbionts within the host cellular arena. Finally, we highlight related research gaps in mosquito virus research.

## Introduction

The term symbiosis, first used by a German botanist, Heinrich Anton de Bary, to describe the living together of fungi and algae as lichens, is today used to describe countless forms of long-term intimate relationships between two species (Relman, 2008; Combes et al., 2018). Such intimate symbiotic associations have since been described in more than half of the animal phyla (McFall-Ngai, 2015). As the appreciation of symbiotic interactions grew, symbionts’ effect on their hosts became increasingly difficult to restrict to beneficial *versus* antagonistic (McFall-Ngai, 2015). Although some mutually beneficial symbiotic interactions exist, the nature of most symbiont-host relationships are not straightforward. Furthermore, even when symbiotic interactions are accepted as mutualistic, they are context-dependent, and the virulence of a symbiont can change, either immediately (i.e. plastically) or over time through evolutionary processes (i.e. selection, genetic drift), due to variations in abiotic and biotic factors (Alizon et al., 2009; Keeling and McCutcheon, 2017).

While research into symbiosis grew rapidly and included a wide range of interactions, it mainly focused on bacteria. Until recently, viruses were often seen solely as causative agents of disease and were left out of the symbiosis conceptual framework (Roossinck and Bazán, 2017). One of the main reasons for this is the inherent bias caused by the available discovery methods for viruses. For bacteria the sequencing of the ribosomal genes permitted less biased detection early on. In contrast, viruses, lacking a shared phylogenetic marker, were much easier to detect and study when they resulted in visible pathogenicity. In the last decades, the number of metaviromic studies has grown significantly, and with this, so has our understanding of the diversity of viruses, their ecology and the wide range of environmental conditions they exist in (Breitbart et al., 2002; Breitbart and Rohwer, 2005; Edwards and Rohwer, 2005; Rosario and Breitbart, 2011).

**Figure 1:**
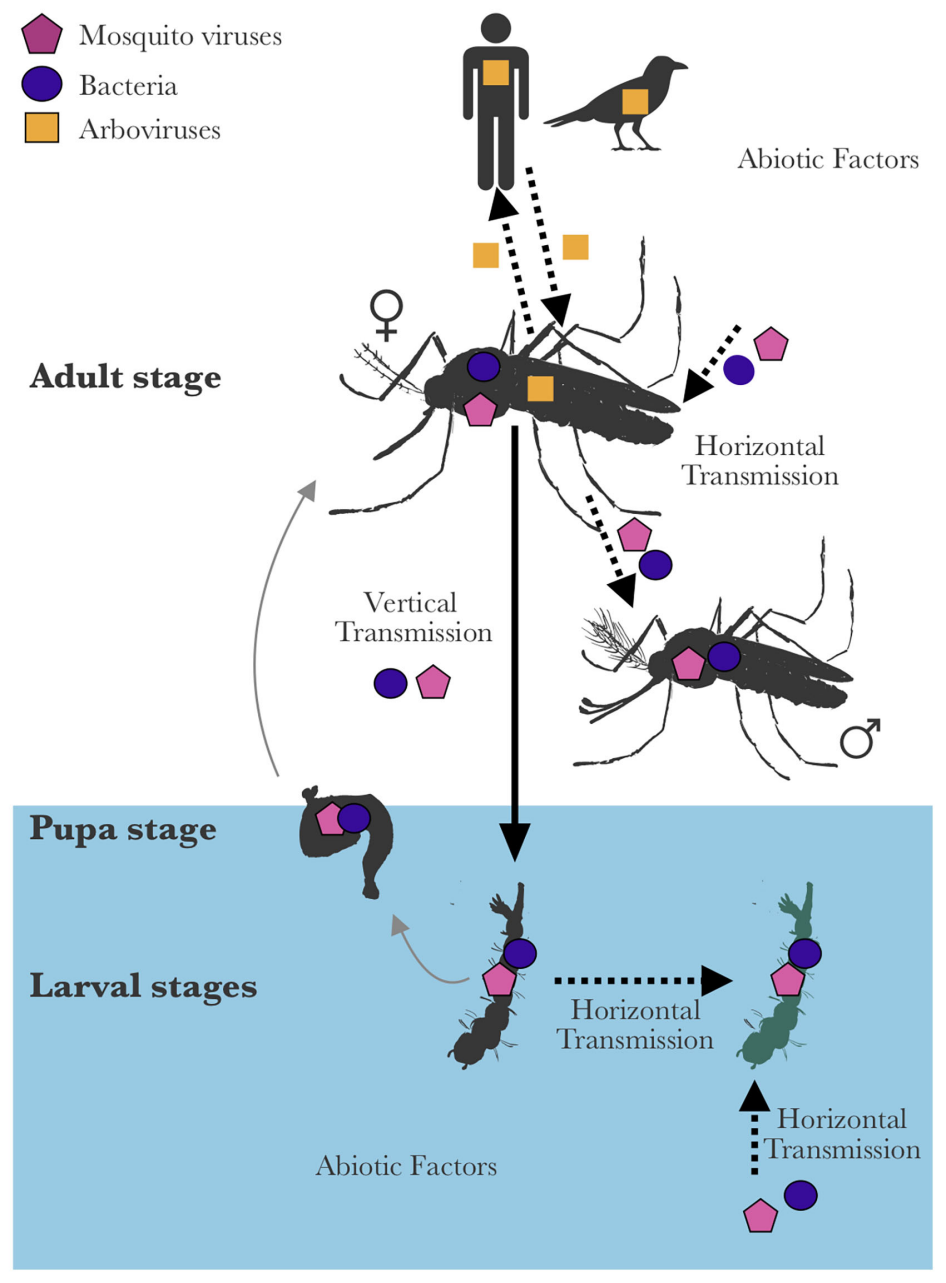
Endosymbiotic interactions of mosquito viruses. Arboviruses are maintained primarily in nature through horizontal transmission and replication cycles in arthropod and vertebrate hosts. Therefore, they primarily infect adult female mosquitoes (arboviruses can also be transmitted vertically, but at very low rates). In contrast, mosquito viruses can be transmitted horizontally (between mosquitoes or from the environment) or vertically (from parents to offspring); and are therefore found in males or aquatic life stages of mosquitoes. They can sometimes be acquired and transmitted with other symbionts. This review discusses the interactions between mosquito viruses and their hosts, the host microbiota, and their place in the host holobiont. The outcome of mosquito virus-mosquito interactions can be context-dependent and influenced by several factors such as transmission routes, host range-host shifts and host immune responses. Interactions between symbionts and symbiont communities can be observed between mosquito viruses and bacteria or other mosquito viruses and also have an impact on the host.

Similarly, due to the limitations of detection tools, earlier studies on mosquito-associated insect-specific viruses (mosquito viruses) were highly inclined towards viruses with easily observable pathology. For instance, DNA viruses from *Baculoviridae* and *Iridoviridae* families were discovered due to the visible symptoms they cause in mosquito larvae; such as hypertrophied nuclei in midguts or iridescence, respectively (Becnel and White, 2007). Following the advances in viral metagenomics, there has been an increase in the number of viruses discovered in natural mosquito populations and mosquito-derived cell lines (Atoni et al., 2019). In particular, the number of mosquito RNA viruses that are phylogenetically related to arthropod-borne viruses (arboviruses) of medical and veterinary importance has grown significantly (Junglen and Drosten, 2013; Blitvich and Firth, 2015; Bolling et al., 2015; Vasilakis and Tesh, 2015; Hall et al., 2016; Halbach et al., 2017; Öhlund et al., 2019). Notably, these circumstances around their discovery led to different approaches to study the DNA and RNA mosquito viruses. DNA viruses have been mainly studied for their pathogenesis during larval stages, while RNA viruses were studied mainly for their interactions with arboviruses during adult stages. The latter have often been called “Insect-specific viruses” to differentiate them from related arboviruses, although ISV literature often excludes mosquito DNA viruses. Nevertheless, both mosquito DNA and RNA viruses can be present in all mosquito life stages (Sang et al., 2003; Saiyasombat et al., 2011; Bolling et al., 2012; Haddow et al., 2013; Kawakami et al., 2016; Ajamma et al., 2018) and are highly prevalent in natural populations (Farfan-Ale et al., 2009; Goenaga et al., 2014; Parry and Asgari, 2018a; Altinli et al., 2019a), suggesting their long-term symbiotic interactions with their hosts (**Figure 1**). These interactions can directly affect the ecology and evolution of the mosquito host or the rest of the mosquito microbiota. Throughout this review, we use the term “mosquito viruses” to refer to all DNA and RNA viruses that are found in mosquitoes, and that cannot infect vertebrates. The term “arboviruses” refers to human and veterinary pathogens transmitted by mosquitoes and infecting both vertebrates and mosquitoes.

Given their intimate and long-term associations with their hosts, we argue that both RNA and DNA mosquito viruses should be studied as mosquito endosymbionts. To date, mosquito virus studies have mostly been focused on i) virus discovery, ii) larval biocontrol or iii) interactions with arboviruses. As the viruses are too often seen as pathogens transiently introduced into a symbiotic system (the mosquitoes and their microbiota), mosquito viruses’ long-term association with their hosts and range of their interactions (i.e. beneficial to antagonistic) are understudied. Thus, investigating mosquito viruses as endosymbionts can improve our understanding of their long-term associations, as well as the wide range of interactions with their hosts and hosts’ microbiota. For instance, several mosquito viruses persistently infect mosquitoes through larval and adult life stages, although whether the infection is actively maintained or merely tolerated by the host immune system is unknown. Similarly, several mosquito viruses can be transmitted vertically, although whether they are fixed in populations or stable on evolutionary timescales is not well investigated. Like other endosymbionts, the interactions between mosquito viruses and their hosts can be diverse, and the outcomes of these interactions may be context-dependent, as shown for a plant virus that confers drought tolerance to its hosts when evolved in drought conditions (González et al., 2021).

Here, we review current knowledge of the interactions between mosquito viruses and their hosts and other symbionts sharing the same cellular arena. The factors (transmission routes, host range-host shifts and host immune response) that influence the outcome of these interactions are presented (**Figure 1**). Finally, to highlight the unknown ecological and evolutionary aspects of mosquito virus interactions, we examine examples of viruses and bacteria found in insects as potential guidance for future research that can improve our understanding of mosquito viruses. A deeper understanding of endosymbiotic interactions between mosquito viruses and their hosts can increase our knowledge of virus ecology and evolution, and inform future vector control strategies.

## Interactions Between Mosquito Viruses and Their Hosts

Similar to bacterial endosymbionts, mosquito viruses could, in principle, harbor both beneficial and antagonistic traits (Zug and Hammerstein, 2015). As mentioned above, many DNA viruses have been discovered due to mass mortality events or visible pathologies during larval stages. Hence, DNA mosquito viruses are often considered highly pathogenic and are studied primarily during larval stages. However, the pathogenicity of viruses can have a wide range even within the same virus family. For instance, densoviruses *(Parvoviridae)* known for their high pathogenicity (Gosselin Grenet et al., 2015) include highly fatal Aedes albopictus densovirus-2 (AalDV2), leading to up to 95% mortality (Perrin et al., 2020), as well as Anopheles gambiae densovirus or Culex pipiens densovirus (CpDV) that cause very low mortality to mosquitoes (Ren et al., 2014; Altinli et al., 2019b).

Many mosquito RNA viruses [with the exception of some dsRNA viruses responsible for apparent pathology (Shapiro et al., 2005; Becnel and White, 2007)] infect their hosts persistently without causing any visible pathology and are often considered commensal. Although these viruses do not seem to cause mass mortality, possible fitness costs are not yet thoroughly investigated. The virulence of mosquito viruses could be manifested in other forms that are much more difficult to detect, such as behavioral changes. For instance, Culex flavivirus (CxFV, *Flaviviridae*) decreased naturally infected *Cx pipiens* mosquitoes’ flight behavior (Newman et al., 2016). However, it is unknown whether this was an indirect consequence of viral replication, leading to a reduction in energy or depletion of flight muscles, or more specific nerve damage that directly affects behavior. Interestingly CxFV has been found in the heads of infected mosquitoes, making the latter hypothesis plausible (Kent et al., 2010; Saiyasombat et al., 2011). This alteration of flight behavior can interfere with the host-seeking behavior of the mosquito, resulting in a decrease in fitness.

Despite the abundance of mosquito virus infections without mass mortality, no beneficial virus infections have been documented so far in mosquitoes, in contrast to other groups of insects. For example, parasitoid wasps harbor a beneficial vertically transmitted entomopoxvirus that ensures the development of the wasp larvae within the wasp’s insect host (Coffman et al., 2020). Insect viruses with beneficial traits have also been shown in lepidopteran species where a vertically transmitted Helicoverpa armigera densovirus increases developmental rates, lifespan, female fecundity, and more importantly resistance against pathogenic *Bacillus thuringiensis* and a baculovirus in laboratory conditions (Xu et al., 2014; Xiao et al., 2021). Hence, this densovirus can be beneficial to its host, specifically in the areas where *B. thuringiensis* or baculovirus based population control tools are commonly deployed (Xiao et al., 2021). Another conditional beneficial interaction has been reported between the rosy apple aphid and Dysaphis plantaginea densovirus (DplDV) (Ryabov et al., 2009) which causes the appearance of wings in a proportion of genetically identical clonal aphids in poor diet conditions (Ryabov et al., 2009). These winged aphids are smaller and have lower fecundity than their wingless counterparts, but can be necessary for the survival of the clonal population when the host plant is not viable, as DplDV free aphids cannot disperse to new plants (Ryabov et al., 2009).

Abiotic factors (e.g. temperature or water pH) can influence the outcome of host-virus interactions through their effect on the individual host (e.g. host immunity) or host populations (e.g. population density), or alternatively by altering the infectivity of the virus. The effect of abiotic factors on mosquito virus infections has been mostly studied for dsRNA and DNA viruses of mosquitoes during larval stages, but little to no information is available for RNA viruses. Infectivity of mosquito baculoviruses and reoviruses can depend on ion composition in the rearing water (Becnel et al., 2001; Shapiro et al., 2004; Shapiro et al., 2005; Green et al., 2006; Becnel and White, 2007). Temperature can also play an essential role in infection, as seen for Aedes albopictus densovirus, where the mosquito larvae infection rate decreased at both lower or higher temperatures than the optimal temperature of 28°C (Li et al., 2019). Abiotic factors can also affect host-virus interactions indirectly through changes in the host population. For instance, AeDV (Thai strain) prevalence in *An. minimus* larvae correlates positively with rainfall (two months prior to larval collection), but negatively with AeDV prevalence in adults, in the following month (Rwegoshora et al., 2000). Thus, it can be hypothesized that rainfall-related changes in the larval population can affect infection dynamics, possibly leading to a higher mortality for infected larvae which may eventually cause a lower prevalence in adult mosquitoes.

Overall, the nature of mosquito virus interactions with their hosts may be context-dependent and could be influenced by various biological and ecological factors such as transmission routes, host range, and host antiviral immunity (**Figure 1**). These factors are often interconnected and can change the immediate outcomes of the host-virus interactions or possibly drive virulence evolution over time (**Table 1**).

**Table 1:** Summary and open questions.

	What is known	Open questions
**Biology**	The gradient of the interactions: costly, neutral or beneficial. Mostly costly interactions were studied for mosquito viruses, although there are examples of beneficial features for insect viruses. The cost of ‘persistent’ infections is not known.	Can mosquito viruses confer benefits on their hosts? Do ‘persistent’ infections have a fitness cost? Does virulence change with different conditions and developmental stages?
	Endosymbionts can be tolerated or actively controlled by the host immune system. Endosymbionts can also impact the host immune system (maturation of humoral and cell responses (apoptosis), gene expression change). Intimate interactions between mosquito viruses and host innate immunity interactions have been demonstrated with the host RNA interference response.	Does the host tolerate or actively control mosquito viruses? What host immune responses do mosquito viruses trigger in addition to RNA interference? Can mosquito viruses modulate host immunity? Are there other immune pathways that play a role in mosquito-mosquito virus interactions in addition to RNA interference?
**Ecology**	Endosymbionts can be highly prevalent, sometimes fixed in host populations in case of mutualistic symbionts or reproductive parasites. Mosquito virus is variable but can be high in nature.	What is the prevalence of a given mosquito virus in nature, and can it be related to the nature of their interactions with their hosts? What is the host range of a given mosquito virus? Do they exhibit similar phylogenies to their hosts?
	Symbiont-symbiont interactions can shape the host microbiota composition. Mosquito viruses can interact with the host microbiota.	How do mosquito viruses interact with each other and the rest of the microbiota? What are the mechanisms of putative interference and facilitation?
	With the exception of primary symbionts that are exclusively vertically transmitted, endosymbionts may exhibit mixed transmission routes and influence their virulence evolution. The transmission routes of mosquito viruses are not well studied, although mixed transmission seems possible.	Can mosquito viruses switch plastically from vertical to horizontal transmission? What are the outcomes of the transmission routes in their virulence? How can abiotic factors or interactions with the rest of the microbiota affect the transmission of mosquito viruses?
	Bottlenecks can occur during transmission or colonization of a given tissue.	How is the evolution of the mosquito viruses shaped by different transmission routes or by tissue tropism?
**Evolution**	The holobiont concept: important for vertically transmitted symbionts. First characterization of the core virome in mosquitoes suggests that viruses could be considered part of the host holobiont.	What are the factors shaping this core virome? How stable is the core virome? How does the core virome of different species affect their hosts and host vector competence? Is phylosymbiosis a common pattern in mosquito viruses and their hosts?
	Endosymbionts often exhibit insertion of partial or complete genomes in host genomes. Endogenous viral elements related to mosquito viruses are found inserted in mosquito genomes.	How common are the functional endogenous viral elements? What could be their function?

### Transmission Routes of Mosquito Viruses

#### The Importance of Transmission Routes in Symbioses

Symbionts can be transmitted to new hosts by horizontal or vertical transmission routes (Bright and Bulgheresi, 2010). These transmission routes play a defining role in the ecology of symbionts (i.e. their spread and their maintenance in nature), as well as in the evolution of their virulence (Cressler et al., 2016). Vertical transmission occurs from parents to offspring. For most endosymbionts, maternal vertical transmission is commonly observed and expected to lower their virulence (except for reproductive parasites) as the fitness of the endosymbiont is related to female fitness. Maternal vertical transmission can occur through the oocytes (transovarial transmission) or on the egg’s surface (transovum transmission) (Fine, 1975). For viruses, vertical transmission can also be paternal (e.g. through sperm) or bi-parental as observed for drosophila sigma- and partitiviruses (Longdon and Jiggins, 2012; Cross et al., 2020). Contrary to single parent vertical transmission, bi-parental vertical transmission does not necessarily lead to reduced virulence as the virus could still spread through the host population despite causing a fitness cost. For instance, although mainly bi-parentally vertically transmitted, Drosophila melanogaster sigmavirus reduces female fertility and slows down host development (Fleuriet, 1981; Longdon et al., 2012). Horizontal transmission routes include venereal transmission, environmental transmission, contact transmission and vector transmission. In contrast to vertically transmitted symbionts, horizontally transmitted symbionts are more likely to evolve towards antagonism as their fitness is not directly linked to the host fitness (Cressler et al., 2016).

#### Mosquito Viruses With an RNA Genome

Arboviruses are mainly maintained in nature by horizontal transmission cycles between vertebrate and arthropod hosts. Many mosquito viruses with RNA genomes are phylogenetically related to arboviruses, although they lack a vertebrate host. In the absence of this obvious horizontal transmission cycle, these mosquito viruses were generally assumed to be transmitted vertically. However, experimental evidence for such vertical transmission is rare (Bolling et al., 2011; Saiyasombat et al., 2011; Bolling et al., 2012; McLean et al., 2020; Ye et al., 2020). For instance, *Cx. pipiens* transmit the CxFV efficiently to their offspring when the mosquitoes were infected naturally but not when the virus was injected experimentally. These results demonstrated that vertical transmission is possible, although dependent on the infection methods for CxFV (Bolling et al., 2011; Saiyasombat et al., 2011; Bolling et al., 2012). Notably, whether this parent to offspring transmission is true transovum or transovarial maternal vertical transmission or happens by contact between parents and offspring has not yet been studied. For CxFV, the role of paternal transmission is not clear either, although for a partitivirus (dsRNA virus) efficient bi-parental transmission has been shown in *Ae. aegypti* mosquitoes (Cross et al., 2020).

In some cases, mosquito virus presence in male mosquitoes was interpreted as evidence of vertical transmission, as it proves that the virus was not only transmitted horizontally to adult females through blood-feeding (Hoshino et al., 2007; Farfan-Ale et al., 2010). However, caution must be taken when interpreting these results, as horizontal transmission from the environment or other mosquitoes during different life stages can also be responsible for mosquito virus presence in males, larvae or eggs. Horizontal transmission can occur either during adulthood (through food, environment, or venereal transfer between adults) or through larval stages (**Figure 1**). To date, horizontal transmission *via* food sources in adult mosquitoes has only been tested through blood-feeding for a handful of viruses belonging to the flavivirus, negevirus, alphavirus and mesonivirus genera. Transmission through infected blood meals was not successful for the tested flaviviruses (CxFV, Palm Creek virus) (Kent et al., 2010; Hall-Mendelin et al., 2016) but was possible for an alphavirus (Eilat virus) and a mesonivirus (Yichang virus), in case of high virus titres (Vasilakis et al., 2013; Nasar et al., 2014; Ye et al., 2020). Mosquitoes can also be horizontally infected during larval stages, although only a few studies have been performed for RNA viruses. For instance, Kamiti River virus (KRV, *Flaviviridae*) and the Yichang virus can infect larvae when added to rearing water; although this was not the case for CxFV (Lutomiah et al., 2007; Bolling et al., 2012; Ye et al., 2020). However, the same strain of CxFV has been found in *Cx. pipiens* and *Cx. tritaeniorhyncus* that do not hybridise but share the same habitat, suggesting the occurrence of horizontal transmission between these species in natural populations (Obara-Nagoya et al., 2013).

#### Mosquito Viruses That Were Mainly Studied in Larval Stages

Studies into DNA (iridoviruses, baculoviruses and densoviruses) and dsRNA (reoviruses) mosquito viruses have focused primarily on horizontal transmission during the larval stages (**Figure 1**) due to their potential use as biological mosquito control tools in larval habitats (Carlson et al., 2006; Becnel and White, 2007; Johnson and Rasgon, 2018). Indeed, several baculoviruses and reoviruses can infect *Aedes, Culex* or *Uratoaenia* larvae when added to their habitats (Shapiro et al., 2005; Green et al., 2006). A vector can also enhance mosquito virus horizontal transmission. For instance, the Mosquito Iridescent virus is transmitted by *Strelkovimermis spiculatus*, a nematode parasitizing *Cx. pipiens* larvae (Muttis et al., 2013; Muttis et al., 2015). On the other hand, mosquito DNA viruses can be vertically transmitted if mosquitoes survive the initial infection. For instance, following Aedes albopictus densovirus infection, surviving infected *Aedes aegypti* larvae can emerge and transmit densovirus to their offspring with varying efficiency (28%-55%) depending on the virus titre in females (Barreau et al., 1997). Another densovirus, Cx. pipiens densovirus (CpDV), can also be transmitted transovarially at a low rate in naturally infected laboratory colonies (Altinli et al., 2019b). In addition, the titre of virus in the ovaries and the rate of vertical transmission is reduced following antibiotic treatments suggesting an effect of the microbiota on CpDV transmission (Altinli et al., 2019b).

Although exclusive vertical or horizontal transmission cannot be excluded, to date, studies suggest that mixed-route transmission, including both horizontal and vertical transmission routes, is likely key to mosquito virus persistence and dispersal in nature. Each transmission route’s role for a given host-virus combination may change depending on the ecological context. Although not explicitly studied for mosquito viruses, abiotic conditions that affect the host population density could also cause a switch between transmission routes, as horizontal transmission may play a greater role in high population density than low population density (Ebert, 2013). Furthermore, biotic factors, such as the microbiota of the mosquito or the abundance of the virus in the larval habitat, can influence virus transmission. However, the exact role of different transmission routes and conditions that can cause a switch from one modality to another are not well understood (**Table 1**).

### Host Range and Host Shifts

The host range of mosquito viruses is not well studied, and for many, it is difficult to define an original host. Particularly for mosquito RNA viruses related to arboviruses (e.g. *Flaviviridae, Togaviridae* and *Bunyavirales*), host range studies have focused solely on their inability to infect vertebrates (Nasar et al., 2012; Junglen et al., 2017; Elrefaey et al., 2020). Even this inability to infect vertebrate cells has only been determined for a handful of mosquito viruses, while the majority of them have been categorized as such, only based on their phylogenetic proximity to other mosquito viruses.

Mosquito viruses are usually named after the mosquito species in which they were discovered. However, it has to be kept in mind that this mosquito species is not necessarily the original or the only host species of the virus. *In vivo* studies are often restricted to species where the virus strain was first reported and their close relatives, although some viruses can have wider host ranges (i.e. other insects or mosquito genera). For instance, Aedes aegypti densovirus (AeDV) and Aedes albopictus densovirus (AalDV), isolated from *Ae. aegypti* and *Ae. albopictus*, respectively, can infect *Aedes*, *Culex* and *Culiseta* species but not *Anopheles* species or other insects tested (Carlson et al., 2006). Other mosquito viruses have a narrower host range, such as Parramatta River virus, which infects several *Aedes* cells lines but not those derived from *Anopheles* or *Culex*. Similarly, An. gambiae densovirus (AgDV) infects only *An. gambiae* but not *An. stephensi* (McLean et al., 2015; Suzuki et al., 2015).

The virulence of mosquito viruses may vary between different mosquito species, particularly between “original” and “naïve” hosts, perhaps due to maladaptation as commonly observed for emerging diseases (Weiss, 2002). For instance, Negev virus can infect and replicate in cell lines derived from *Ae. albopictus*, *An. albimanus, An. gambiae, Cx. tarsalis* but not *P. papatasi*, and *D. melanogaster*. Interestingly, this virus only caused a cytopathic effect (CPE) in *Ae. albopictus* and *Cx. tarsalis* cell lines. However, CPE was not observed in *An. albimanus*, where the virus replicates successfully (Vasilakis et al., 2013). In this case, higher virulence could be the result of an introduction to a new host and, therefore, suggests that *An. albimanus* is the original host. In this context, studying the epidemiology and phylogeny of mosquito viruses in nature, combined with laboratory experiments, would be helpful to understand viral host range and the effect of host shifts on the virulence and transmission route evolution.

Mosquito viruses could shape host distribution in nature through host specific virulence. A good example of this, is a study conducted in Thailand where *Ae. aegypti* adults showed a high (44.3%) Aedes densovirus (AeDV, Thai strain) prevalence while all tested *Ae. albopictus* adults were negative (Kittayapong et al., 1999). In contrast, experimental larval infections showed that AeDV could infect both species of mosquitoes and was more lethal to *Ae. albopictus* than to *Ae. aegypti* larvae. One hypothesis is that the high virulence of AeDV prevented *Ae. albopictus* larvae development into adults in natural populations, resulting in a lower prevalence in adults of this species. If so, this suggests that AeDV infection may relax competitive pressure for *Ae. aegypti* larvae, thus contributing to *Ae. albopictus* and *Ae. aegypti* population distribution in nature (Kittayapong et al., 1999).

### Insect Immune Response Against Mosquito Viruses

One of the most important factors defining the outcome of mosquito virus-host interactions is the host immune response. The mosquito antiviral immune response has been extensively studied in the context of arboviruses with an emphasis on the RNA interference (RNAi) pathway as it acts antiviral against all investigated arboviruses, to date (Donald et al., 2012). RNAi is a sequence-specific silencing mechanism that works through the production of virus-derived RNAs resulting from the cleavage of viral replication intermediates. Depending on the size of the RNA molecules and the host proteins involved in their production, these small RNA molecules are called: siRNAs (21 nts) or piRNA (24-30 nts) (Leggewie and Schnettler, 2018). Viral (v)siRNA or (v)piRNAs are taken up by a protein complex and used as a guide to find complementary viral RNA sequences, to initiate their degradation.

All studies, to date, have shown that mosquito virus-derived small RNAs are produced, proving an interaction between the mosquito RNAi response and mosquito viruses (for both DNA and RNA viruses) (Agboli et al., 2019). It is not yet known whether RNAi is antiviral against mosquito viruses, but it is hypothesized that a delicate balance between the RNAi defense and the virus counter defense is responsible for the persistent infection state observed for many RNA mosquito viruses. Several mosquito and other insect-specific viruses have indeed been shown to produce proteins or other molecules that interfere with the antiviral RNAi response as a counter defense, called “viral suppressors of RNAi” (Chao et al., 2005; Schuster et al., 2014; van Cleef et al., 2014). Persistent infection often lacks high mortality or visible pathology in infected cells and is manifested by fluctuating viral titres (Franzke et al., 2018; Bishop et al., 2020). Any disruption of this balance can change the virulence of the virus. For example, Flock House virus (FHV) infection in drosophila is normally non-pathogenic; however, its pathogenicity increases when FHV infected flies lack Argonaute 2, a key protein of the siRNA pathway (van Rij et al., 2006).

In addition to encoding RNAi suppressors, mosquito viruses can modulate mosquito immunity through the integration of viral genetic material into the host genome. Many endogenous viral elements (EVEs) related to mosquito viruses have been reported from mosquito genome sequences (Katzourakis and Gifford, 2010; Palatini et al., 2017). EVEs originating from RNA viruses, called non-retroviral integrated RNA elements (NIRV), closely related to mosquito viruses from *Bunyavirales, Reoviridae, Rhabdoviridae* and *Flaviviridae* have been found in the genome of a variety of mosquito species. NIRVs in mosquitoes have been linked to the piRNA pathway, as mosquito virus-derived NIRVs were mostly found in piRNA clusters in mosquito genomes and the production of NIRV-specific piRNAs was also shown (Whitfield et al., 2017; Varjak et al., 2018). Moreover, the antiviral activity of these NIRVs-specific piRNAs was recently established in mosquito-derived cells and mosquitoes (Tassetto et al., 2019; Suzuki et al., 2020). In both cases, piRNAs derived from a Cell Fusing Agent virus (CFAV)-specific NIRV were able to (i) inhibit CFAV replication specifically in the ovaries of *Ae. aegypti* mosquitoes, (ii) reduce the expression of a reporter construct harboring CFAV-specific NIRV target sites or (iii) inhibit the infection of a virus harboring CFAV-specific NIRV target sites. These data support the hypothesis that the acquisition of NIRVs, at least in the mosquito genome, acts as an adaptive immune response. Such integration of mosquito virus sequences can occur in somatic or germ cells, but only the latter can be transmitted vertically to the offspring. Therefore, tissue specificity of mosquito viruses strongly influences vertical NIRV transmission, hence the acquisition and transmission of this “adaptive immune response”. Moreover, a comparison of different mosquito genera, sampled from the same habitats suggests that NIRV integration is not just a mere result of virus exposure but also depends on specific virus-host interactions (Palatini et al., 2017). The majority of NIRVs are found in *Aedes* mosquitoes, specifically *Ae. aegypti* (Palatini et al., 2017). Until now, the reason for this is unknown, but a possible explanation could include differences in the immune response, susceptibility to certain viruses that are more prone to NIRV production, and differences in the presence of retrotransposons (as these are essential for NIRV production) and microbiota.

There are still many unanswered questions about how this would affect a virus infection in nature, the extent to which this “adaptive” immunity shaped the virus evolution, and the frequency of the functional EVEs derived from mosquito viruses (**Table 1**). In addition, mosquito virus interactions with other aspects of mosquito immune system are also yet to be investigated.

## Symbiont-Symbiont Interactions

**Figure 2:**
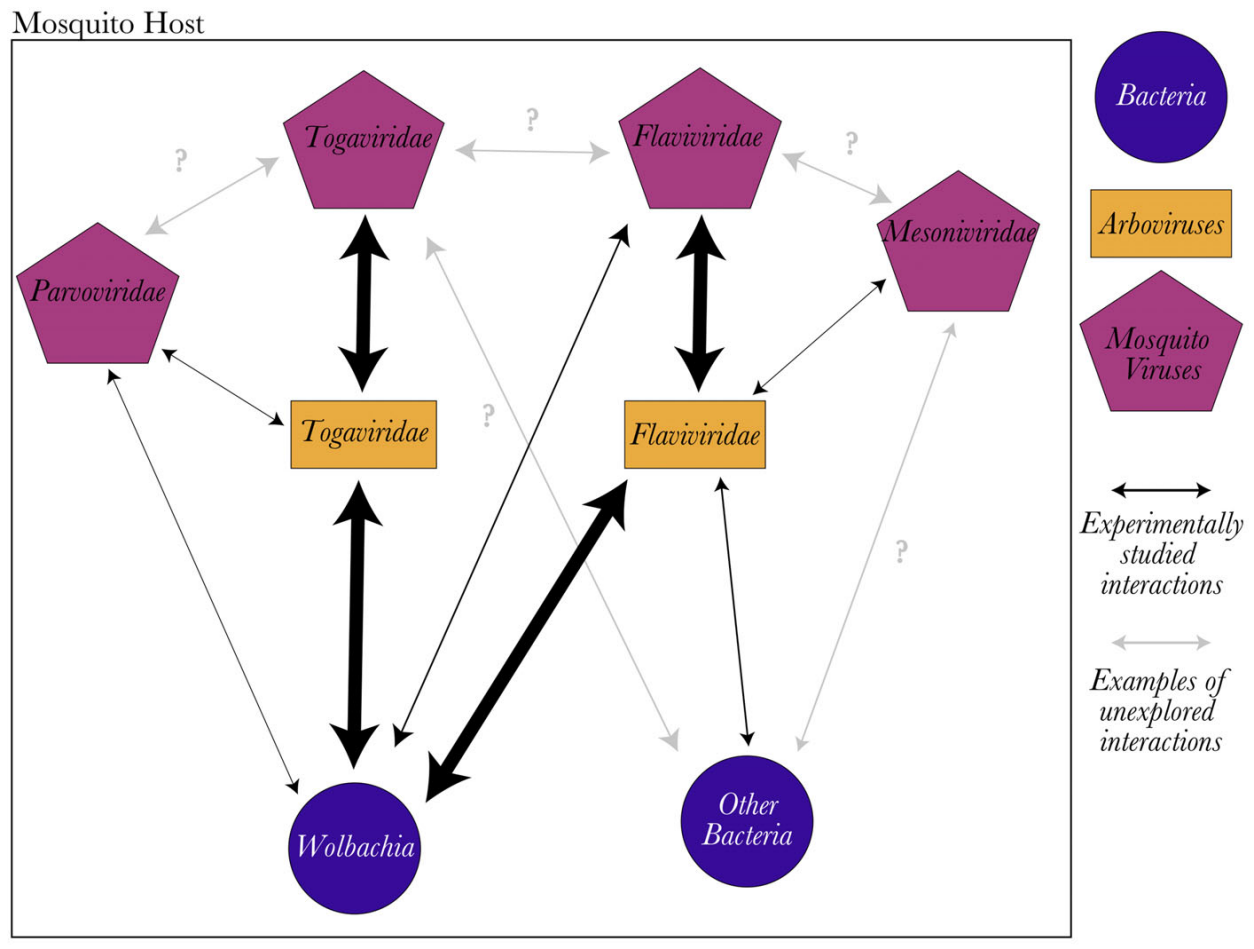
Mosquito virus interactions with the host microbiota. Most experimental studies of microbiota interactions have focused on arbovirus inhibition by bacterial or viral symbionts in important mosquito vectors (represented as wide arrows). However, the rest of the interactions between microbiota and mosquito viruses were poorly studied (represented as thin arrows). Many possible interactions have never been explored experimentally (grey arrows indicate some examples), and additional studies are needed to understand the outcomes of these interactions on mosquito host and host vector competence. Nothing is known about mosquito-virus interactions with each other or the possible mechanisms of these interactions.

While host-symbiont interactions are often studied in a binary manner, symbiont-symbiont interactions can also determine infection outcomes. In mosquitoes, studies on microorganism interactions have been focused on mosquito endosymbiont interference with arboviruses (**Figure 2**), for example *Wolbachia* whose interactions could prove useful for arbovirus control (Flores and O’Neill, 2018). *Wolbachia* natural infections are common and sometimes fixed in mosquitoes, including several arbovirus vectors such as *Aedes albopictus* and *Culex pipiens* (Sicard et al., 2019). Furthermore, it is possible to create mosquito lines stably transinfected with *Wolbachia* from other mosquito species or drosophila (Hughes and Rasgon, 2014). In these transinfected mosquitoes, *Wolbachia* reduces the infection rate and virus load of important arboviruses (Moreira et al., 2009; Walker et al., 2011; Hussain et al., 2012; van den Hurk et al., 2012; Johnson, 2015; Pimentel et al., 2021). This success of *Wolbachia* to interfere with arboviruses, brought attention to the rest of the microbiota. The presence of other bacterial symbionts in addition to *Wolbachia*, especially in the gut, has been characterized both during adult and larval stages (Caragata et al., 2019). Although the overall bacterial community depends on environmental conditions, a core microbiome has been defined for some mosquito species (Guégan et al., 2018). Some of these core bacterial symbionts have been shown to interact with arboviruses (Huang et al., 2020). For example, *Serratia odorifera* enhanced *Ae. aegypti’s* susceptibility to dengue virus (DENV, *Flaviviridae*) through the production of a bacterial protein called *sm*Enhancin. Indeed, *sm*Enhancin has been shown to facilitate viral dissemination from the gut by digesting mucines on the mosquito gut epithelia (Wu et al., 2019).

Because of this promising approach to arbovirus control, studies on RNA mosquito viruses have focused heavily on their interactions with arboviruses. Similar to some bacterial endosymbionts, mosquito viruses can also interfere with arbovirus replication (**Figure 2**) [reviewed in (Agboli et al., 2019; Öhlund et al., 2019)]. Arbovirus interference by mosquito viruses is mainly observed when both belong to the same virus family. For example, Palm Creek virus (*Flaviviridae*) interferes with West Nile virus (WNV, *Flaviviridae*) replication but not with Ross River virus (*Togaviridae*) replication in Aedes-derived mosquito cells (C6/36) (Hobson-Peters et al., 2013). Culex Flavivirus can also reduce the replication of WNV (Bolling et al., 2012), although it did not affect Rift Valley fever virus (*Phenuiviridae*) in *Cx. pipiens* (Talavera et al., 2018). So far, only a mesonivirus (Yichang virus) has been shown to interfere with an arbovirus from a different family (DENV, *Flaviviridae*) *in vitro* and reduce its transmission rate in *Ae. albopictus* (Ye et al., 2020). In contrast to mosquito RNA viruses, DNA viruses have rarely been studied for their interactions with arboviruses. Only Anopheles gambiae densovirus has been shown to interfere with Mayaro virus (*Togaviridae*) infection both *in vivo* and *in vitro* in *An. gambiae* (Urakova et al., 2020).

Arboviruses mainly infect female adult mosquitoes through infectious blood meals although they can be transmitted vertically at very low rates (Lequime and Lambrechts, 2014; Lequime et al., 2016). Unlike arboviruses, mosquito viruses can be found in both female and male mosquitoes at all life stages (**Figure 1**). Since some mosquito viruses can be efficiently transmitted to offspring along with other mosquito endosymbionts, they could have an impact on their respective transmission. The interactions between mosquito endosymbionts (bacteria-bacteria, virus-virus or virus-bacteria) can therefore cause changes in their respective ecology (e.g. increased vertical transmission in the presence of a given symbiont) and hence their interactions with their hosts at evolutionary time scales (e.g. reduction of virulence).

Interactions between different mosquito viruses have not yet been studied. Furthermore, studies on interactions between mosquito viruses and mosquitoes’ bacterial microbiota are limited to their interactions with *Wolbachia* (**Figure 2**). In general, *Wolbachia* appears to facilitate mosquito virus infections, unlike arbovirus infections. For instance, in *Ae. aegypti*-derived cells, *w*AlbB and *w*MelPop enhanced the replication of Aedes albopictus Negev-like virus (*Virgaviridae*) (Bishop et al., 2020) and Aedes anphevirus (Parry and Asgari, 2018b), respectively. A similar positive interaction between *Wolbachia* and Aedes albopictus densovirus (*Parvoviridae*) was observed in *Aedes*-derived cell lines transinfected with *w*MelPop (from *Drosophila melanogaster*) or *w*AlbB (from *Ae. albopictus*), compared to control cells (Parry et al., 2019). No influence of *w*MelPop nor *w*Mel was observed against Phasi Chareon-like virus (*Bunyavirales*) (Schnettler et al., 2016; McLean et al., 2019). In contrast, CFAV (*Flaviviridae*) replication was limited by *w*MelPop, *w*Mel and *w*AlbB in transinfected *Ae. aegypti*-derived cell lines (Schnettler et al., 2016; Zhang et al., 2016; McLean et al., 2019; Bishop et al., 2020). However, released *Ae. aegypti* populations transfected with *w*Mel demonstrated increased abundance of insect-specific flaviviruses (Amuzu et al., 2018).

Interactions between *Wolbachia* and mosquito viruses were also studied in a natural system, where the native *Wolbachia*, the host and a mosquito virus have potentially evolved together. *Culex pipiens (s.l.)* populations that are naturally infected with *Wolbachia w*Pip (Rasgon and Scott, 2003; Dumas et al., 2013; Altinli et al., 2018) also harbor CpDV in high prevalence (Altinli et al., 2019a). In the laboratory, CpDV and *w*Pip can be co-transmitted to the offspring of *Cx. pipiens (s.l.)* lines vertically. CpDV levels in ovaries and its vertical transmission decreases significantly in *w*Pip free mosquitoes compared to *w*Pip infected females (Altinli et al., 2019b). These results suggest that *Wolbachia* can affect the transmission of the mosquito virus and drive its infection dynamics in natural populations. Indeed, a specific strain of *Wolbachia* (*w*Pip-IV) was associated with higher CpDV loads in ovaries of laboratory colonies (Altinli et al., 2019b) and a higher prevalence in nature compared to another *w*Pip type (i.e. *w*Pip-I) (Altinli et al., 2020). *w*Pip induces cytoplasmic incompatibility, a conditional sterility in crosses between females and males infected with incompatible *Wolbachia* strains in *Cx. pipiens (s.l.).* Thus, it is possible that mosquito viruses associated with the more advantageous *w*Pip strain could also invade the host population if vertically co-transmitted.

**Figure 3:**
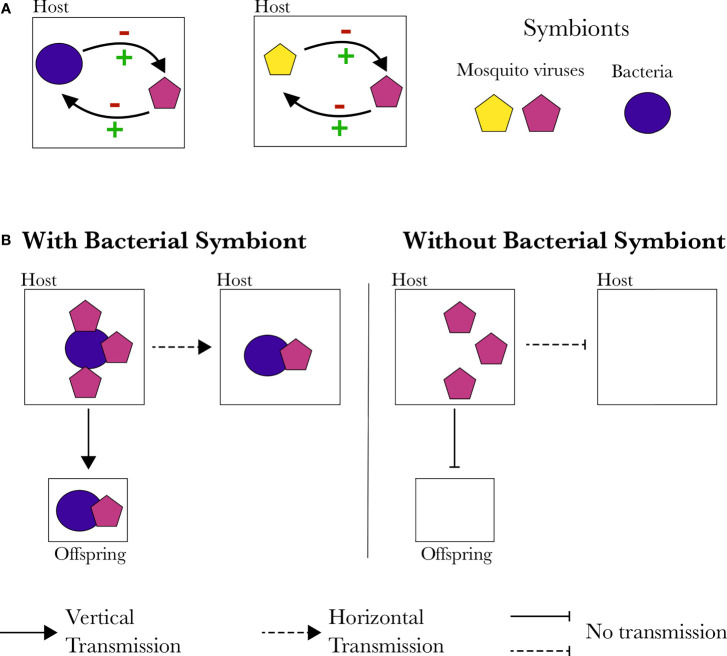
Possible interactions between mosquito endosymbionts. **(A)** Gene products of a symbiont may affect other symbionts. This effect can either be direct or indirect (e.g. through modulation of the host cellular environment or immune responses) resulting in an increase or decrease in entry or replication of another symbiont. **(B)** Direct protein interactions can increase or block other symbionts horizontal or vertical transmission or cell entry.

To date, the molecular mechanisms underlying mosquito virus interactions with the rest of the microbiota, or arboviruses, have not been studied (**Figure 2**). Symbionts can interact with each other in the cellular arena either directly (i.e. direct protein-protein interactions, metabolism connections, resource competition or *via* toxin production) or indirectly through their extended phenotype (i.e. through modulation of the host cellular environment or immune system, or through host reproduction manipulation such as cytoplasmic incompatibility) (**Figure 3**) (Douglas, 2016; Almand et al., 2017; Zélé et al., 2018). The “facilitation” of mosquito viruses by *Wolbachia*, for example, could be the result of increased viral binding and shedding due to direct protein-protein interactions, as demonstrated for poliovirus and mice gut microbiota (Kuss et al., 2011; Moore and Jaykus, 2018). Protein-protein interactions can also facilitate the horizontal and vertical transmission of viruses (**Figure 3**). Although not yet investigated in mosquitoes, bacterial symbionts’ facilitation of virus transmission has been observed in other arthropods. For instance, Rice dwarf virus (*Reoviridae*) can be vertically transmitted to their vector’s offspring by binding to the outer membrane protein of *Sulcia* (an obligate vertically transmitted bacterium of leafhoppers) with its viral capsid protein (Jia et al., 2017). Similarly, horizontal transmission success of the Tomato Yellow Leaf Curl virus (TYLCV*, Geminiviridae*) depends on the presence of *Hamiltonella* bacteria in its whitefly vector (*Bemicia tabaci)*, as the GroEL protein of *Hamiltonella* binds to the capsid of TYLCV, facilitating the transmission to host plants (Gottlieb et al., 2010; Su et al., 2013). Indirect interactions where one of the organisms modulates, for example, the host’s immune response, may also explain the observed facilitations.

## Mosquito Viruses in the Holobiont Context

Extended phenotypes do not solely result from host-symbiont or symbiont-symbiont interactions (*e.g.* “the” pathogen *Vibrio cholerae* or “the” mutualist *Buchnera aphidicola*) like previously thought, but are driven or influenced by interactions between microbial communities in an organism (Vayssier-Taussat et al., 2014). This paradigm shift brought up the “holobiont” (i.e. the host and its associated microbiota) and hologenome concepts. The latter describes the genomes of the host and associated microbiota at a given time, which could act as a unit of natural selection (Zilber-Rosenberg and Rosenberg, 2008). However, the importance of holobiont in evolutionary biology is still debated because hosts and their microorganisms do not always exhibit co-evolution. Therefore, a first step would be to assess which part of the symbiotic community belongs to the hologenome with high heritability from generation to generation (Moran and Sloan, 2015). One way to investigate this is to check the congruence between the phylogeny of the host and its microbial communities.

The evolutionary history of a host explaining the divergence of its microbial community is a phenomenon called “phylosymbiosis” (Lim and Bordenstein, 2020). Previously, studies on phylosymbiosis focused on bacterial microbes, with the exception of viral communities of the parasitic wasp, *Nasonia* (Leigh et al., 2018). *Nasonia* bacterial microbiota and virome exhibited a pattern of phylosymbiosis, primarily driven by bacteriophages hosted by the bacterial microbiota (Leigh et al., 2018). Recent studies in mosquitoes have described a distinct core virome in different species of mosquitoes that were collected in the same region and share the same larval habitat (Pettersson et al., 2019; Shi et al., 2019; Konstantinidis et al., 2021). The potential evolutionary significance of this core virome and whether/how it is maintained by the host is unknown. Further phylosymbiosis studies including bacteria, viruses, and other microorganisms can help answer these questions (**Table 1**).

## Conclusions

The discovery of mosquito viruses has increased exponentially over the past decade, and their interactions with arboviruses have attracted much scientific attention. Nevertheless, many questions related to mosquito virus interactions remain unanswered (**Table 1**). For example, the fitness costs of mosquito viruses on their hosts are not well studied. This may be due to difficulties associated with detecting minor fitness effects in laboratory experiments. Nevertheless, combining experimental and natural population studies could help assess the outcomes of mosquito virus infections. In particular, the study of prevalence, phylogeny and host associations may be helpful in this context. Furthermore, our knowledge is partial regarding DNA and RNA viruses (e.g. DNA viruses have been primarily studied for aquatic larval stages, while RNA viruses have been studied for adult stages) which hinders our understanding of the system as a whole.

Mosquito viruses are often overlooked in studies of the mosquito microbiota, yet their symbiotic interactions with their hosts and the rest of the microbiota could define host fitness and vector competence. As has been observed for other symbionts, the nature of these interactions could be context-dependent. They can shape microbial communities and host populations by influencing infection outcomes and can drive the evolution of the different partners involved.

Nevertheless, due to their medical importance, mosquitoes are already well studied for many arbovirus-related aspects. Current knowledge could be leveraged to achieve a more holistic understanding of the mosquito and associated microbiome, including viruses, which will also contribute to mosquito and arbovirus control. The study of non-model organisms and their bacterial symbionts has advanced our knowledge of host-symbiont interactions in previous years. Mosquito viruses may do the same in the future and change our understanding of virulence and virus ecology and evolution.

## Author Contributions

MA wrote the first draft of the manuscript and made the figures. All authors contributed to the article and approved the submitted version.

## Conflict of Interest

The authors declare that the research was conducted in the absence of any commercial or financial relationships that could be construed as a potential conflict of interest.

## Publisher’s Note

All claims expressed in this article are solely those of the authors and do not necessarily represent those of their affiliated organizations, or those of the publisher, the editors and the reviewers. Any product that may be evaluated in this article, or claim that may be made by its manufacturer, is not guaranteed or endorsed by the publisher.

## Abbreviations

AalDV-2, Aedes albopictus densovirus – 2; CpDV, Culex pipiens densovirus; CxFV, Culex flavivirus; DplDV, Dysaphos planginaea densovirus; CPE, Cytophatic effect; AeDV, Aedes densovirus; RNAi, RNA interference; (v)siRNA, (viral) small interfering RNA; (v)piRNA, (viral) Piwi-interacting small RNAs; EVE, Endogenous viral elements; NIRV, non-retroviral integrated RNA elements; FHV, Flock House virus; CFAV, Cell-Fusing Agent virus.
